# Using Augmented Reality to Motivate Oral Hygiene Practice in Children: Protocol for the Development of a Serious Game

**DOI:** 10.2196/10987

**Published:** 2020-01-17

**Authors:** Susy Nazaré Silva Ribeiro Amantini, Alexandre Alberto Pascotto Montilha, Bianca Caseiro Antonelli, Kim Tanabe Moura Leite, Daniela Rios, Thiago Cruvinel, Natalino Lourenço Neto, Thais Marchini Oliveira, Maria Aparecida Andrade Moreira Machado

**Affiliations:** 1 Department of Pediatric Dentistry, Orthodontics and Community Health Bauru School of Dentistry University of Sao Paulo Bauru Brazil; 2 Educational Technology Section Bauru School of Dentistry University of Sao Paulo Bauru Brazil

**Keywords:** video games, education, dental, user-computer interface, computer simulation, pediatric dentistry

## Abstract

**Background:**

New technologies create possible new ways of action, interaction, and learning which is extremely relevant in the field of oral health education. There is a lack of protocol in using an immersive interactive ludic-educational interface to motivate oral hygiene practice in children by means of augmented reality.

**Objective:**

This study aims to present a protocol on the development of a serious game to motivate oral hygiene practice in children.

**Methods:**

A serious game will be designed by augmented reality techniques to improve toothbrushing effectiveness of children aged 6 to 10 years. The functional structure of this interface is activated by means of movements recognized by Kinect (Microsoft Corp). The toothbrushing technique will be available in the game, enabling the children to execute the movement in the virtual environment. By identifying errors, this game will be tailored to improve the oral health of children by correcting the technique and teaching the user the adequate toothbrushing method. A template analysis will be performed to identify barriers and facilitators in each scenario.

**Results:**

After the implementation of the virtual interactive and immersive panels, enrollment will begin and evaluations will be made by means of questionnaires distributed to participants who interact with the game. Thus, an analysis of the product efficacy will be conducted. The expected outcome will be to obtain a digital instrument to motivate oral hygiene practice and enhance health awareness in children.

**Conclusions:**

The serious game will support the prevention of oral diseases by sharing scientific research in the school environment and community.

**International Registered Report Identifier (IRRID):**

PRR1-10.2196/10987

## Introduction

Teaching processes conducted in virtual environments are becoming more prevalent in order to improve quality of life for the population [1,2]. Increasingly, these virtual environments employ augmented reality (AR) as a learning tool and provide a more realistic experience to the students. AR is composed of simulation, association of virtual reality with physical materials, instruments, and feedback to train students and verify their acquired knowledge on specific subjects. The interaction occurs through object manipulation, aiming at specific targets. AR is popular because of its applicability providing the user with unique experiences and great interaction yet promoting activities that stimulate learning. Studies comparing traditional and simulation technology–based learning showed that students had better knowledge retention indexes with the latter [3].

The recent literature reports the use of artificial intelligence to aid in developing children’s personal skills inside a healthy environment [4]. Interactive interfaces directed to the parts of the human body related to oral health by using AR will share scientific knowledge on dentistry in the community, specifically regarding children [5,6]. Kinect (Microsoft Corp) is an interactive interface innovation designed not only for entertainment but also for other purposes such as health education, making the interaction between person and machine more effective. It allows the user to interact with the device only with gestures and movements, without the need of a joystick. These movements are captured by cameras and sensors, providing real interaction, also allowing users to explore the content with commands activated in a ludic or nonludic way. Kinect has several resources (sound, image, depth, infrared, and movement engine) with a high level of accuracy and synchronicity in one single device. These resources offer a series of innovative possibilities of interaction between users, services, and computing applications. Educational and motivational methods play an important role in informing individuals about oral diseases and changing their hygiene habits, starting as soon as children develop their motoric coordination [4]. This study aims to present a protocol on the development of a serious game (immersive virtual environment using AR) to motivate oral hygiene practice in children.

## Methods

### Interactive Panel Configuration

#### General Features

A serious game will be designed using AR techniques to improve toothbrushing effectiveness in children aged 6 to 10 years. The functional structure of this interface is activated by means of movements and recognized by Kinect. The programming language will be based on C#, which has been greatly influenced by the Java programming language. It is oriented toward objects and considered to be simple, with a great performance. Some C# characteristics:

- Language: low complexity; being projected in a simple way
- Object-oriented: classes, attributes, methods, and objects
- Strongly typed: avoiding attribution errors
- Managed language: all the management contained in the memory is done by runtime via Garbage Collector
- Version control: once assembly is formed, it contains information on the code version, either dynamic link library or executible file

However, for programming purposes, the framework Kinect for Windows SDK 2.0 (Microsoft Corp) will be used. This is a free tool for people who want to develop their own games that has a robust yet friendly interface and allows the development of applications based on Kinect (both for PC and the Xbox 360 Console, also Microsoft Corp) [7]. The toothbrushing technique [8-10] will be available in a game, enabling the children to execute movements in a virtual environment. This game will be tailored to improve the oral health of the children by correcting technique and teaching the user the adequate brushing method after identifying errors [11]. This method is easily trackable via the motion control ludic interface provided by Microsoft.

In the final stages, an open-source framework for mobile development compatible with Android and iOS devices and licensed by Adobe Systems will also be used. However, motion control will not be available in mobile devices. Instead a gesture-based control on the device’s screen will be used to track users’ movements.

#### Connected Devices

- Development suite: Kinect for Windows SDK 2.0 (freeware)
- Unity 3D engine platform
- Audio editors: Audacity and FormatFactory (freeware)
- Kinect Xbox 360 sensor (Microsoft Corp)
- High-definition 3D XL2420T monitors (2; BenQ Corp)
- High-performance desktop computers (2), with Intel Core i7 processor, 32 GB RAM memory, 2TB internal storage, and Nvidia Quadro graphics card
- High-performance notebooks (2): ASUS G74SX-DH73-3D 17.3” display, Core i7 2670QM processor, 12 GB RAM memory, 750 GB HDD + 750 GB HDD internal storage and 3D Vision Kit graphics card
- External hard drive: AirPort Time Capsule (Apple Corp) 2TB sharing station
- Television with ultra-high-definition technology

**Figure 1:**
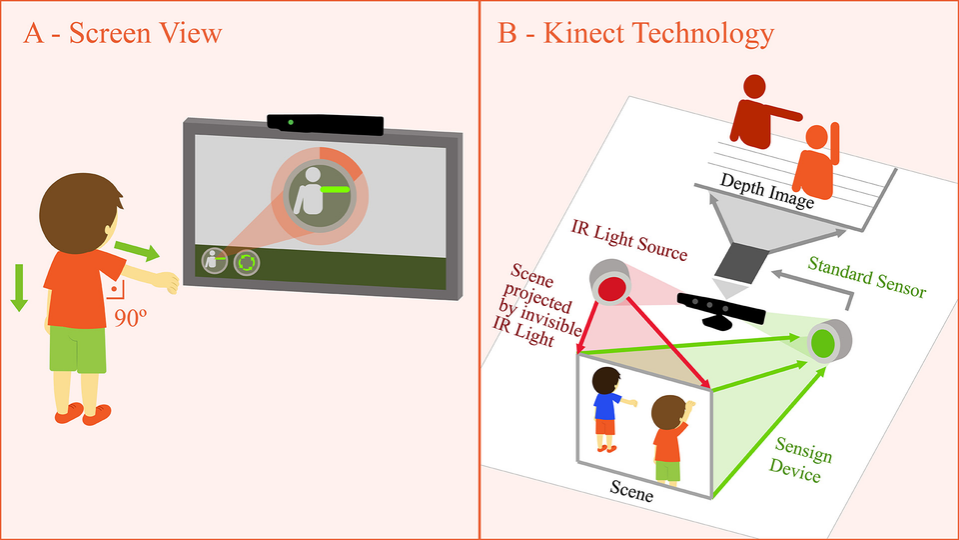
Resources offered by Microsoft’s Kinect device.

An ultra-high-definition television will be used to see the whole game interface and interaction with the Kinect sensor (Figure 1). This process happens hundreds of times per second, thus making the immersion real. This changes the user’s old behavior into a new one, characterized by dynamic and intuitive reactions, stimulating curiosity and creativity.

### Interactive Panel Use

#### Interactive Interface

An interactive interface with an educative instructional aspect results from the intersection of three technological processes: game, computational application, and learning objects [12]. The use of games in school contexts applies the following aspects: (1) anxiety, (2) limits, (3) autonomy, (4) capability of achievement, (5) motor coordination, (6) spatial organization, (7) segmental control, (8) attention and concentration, (9) anticipation and strategy, (10) auditory discrimination, (11) logical reasoning, (12) creativity, and (13) figure and depth perception. In this context, the game is a very appropriate learning tool because it uses its power to attract the interest of students and creates social and personal experiences, triggering new discoveries and maturing their personalities [13]. Furthermore, by enabling innovative and attractive educational practices, these educational interfaces can become important accessories to the process of teaching and learning. The AR interface allows the user to see the real world together with virtual objects [14], consisting of a serious game where the player controls and executes actions by means of corporal movements. The body movements are interpreted as data entry and associated with specific commands to the game, transforming the tridimensional movement of the physical space into an entry into a computational system.

#### Game General View

**Figure 2:**
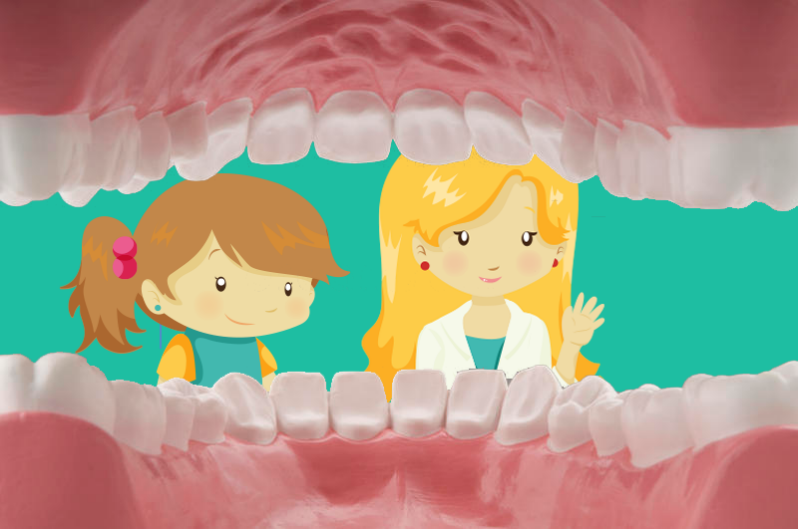
Users’ representation by game characters.

In the construction of the game prototype, the technology employed involves virtual reality combined with the Kinect device. Through sensors, Kinect enables a more efficient interaction between the child and the game, allowing a ludic and immersive interaction in topics related to oral health (Figure 2). This is an essential and important issue for the prevention of dental caries and other related oral diseases.

#### Interface Elements

**Figure 3:**
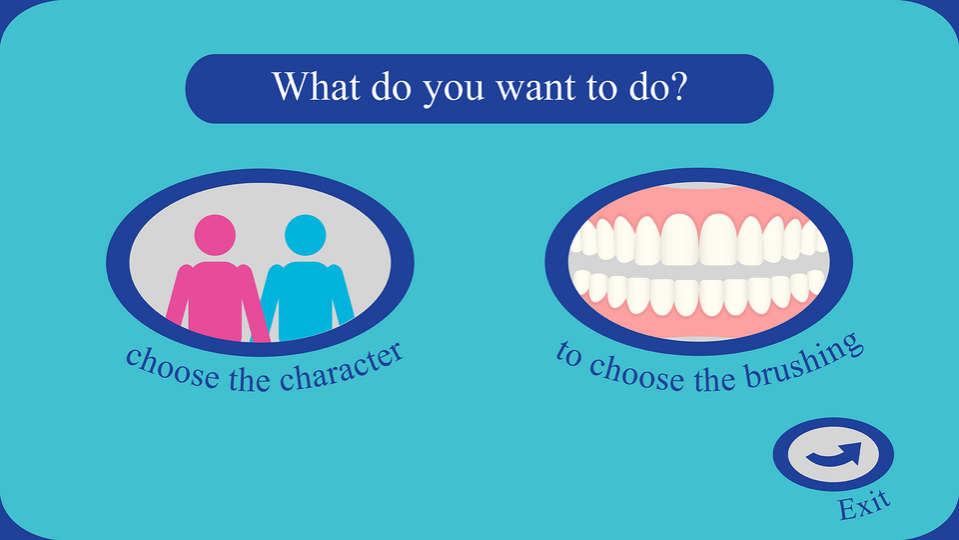
Opening screen of the digital interface with usage options.

**Figure 4:**
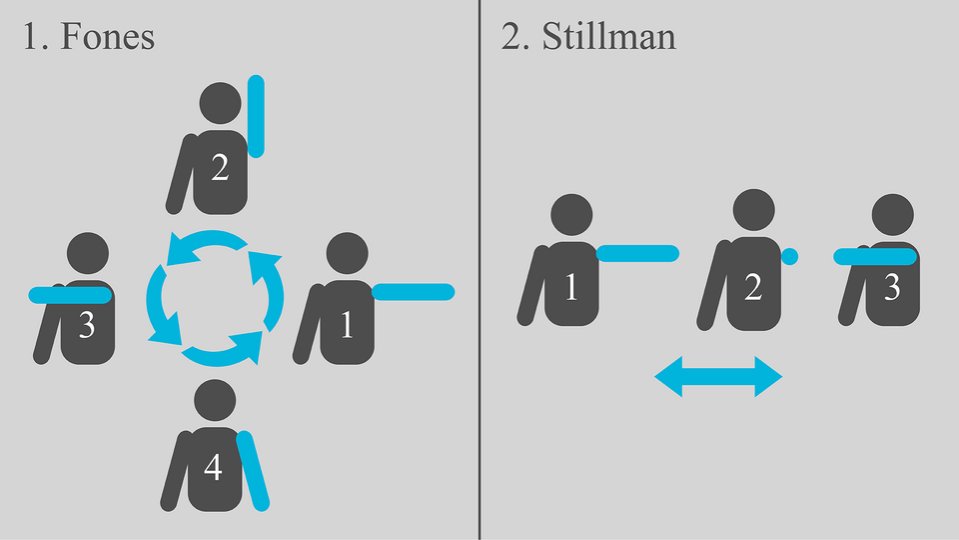
Movement simulations of the proposed techniques (Fones and Stillman).

The opening screen (Figure 3) shows three options to choose from: character, toothbrushing techniques (Fones or Stillman), and the option to quit. In the character option, the player can choose according to gender (male/female). In the toothbrushing option, the player can choose the technique and the training goal (Figure 4).

#### Movement Detection and Error Identification

For movement detection, it will be necessary to (1) habilitate the capture of the human skeleton by the Kinect device, (2) create a code to establish connection between the device and software, (3) create an array (vector) of the skeleton type, attributing data to the vector, (4) verify the vector is being monitored, (5) attribute a variable to the body part to be monitored (in this case, the hands), and (6) create the desired modification (ie, a desirable response whenever the movement executed by the player’s hands is closer to the movements expected in the chosen brushing technique).

**Figure 5:**
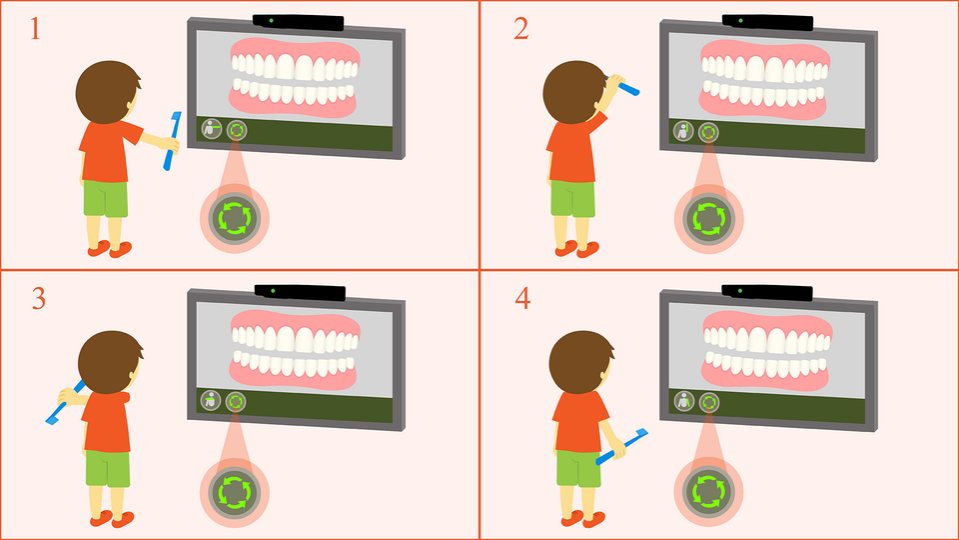
User interaction in the serious game with Kinect using the Fones brushing technique.

**Figure 6:**
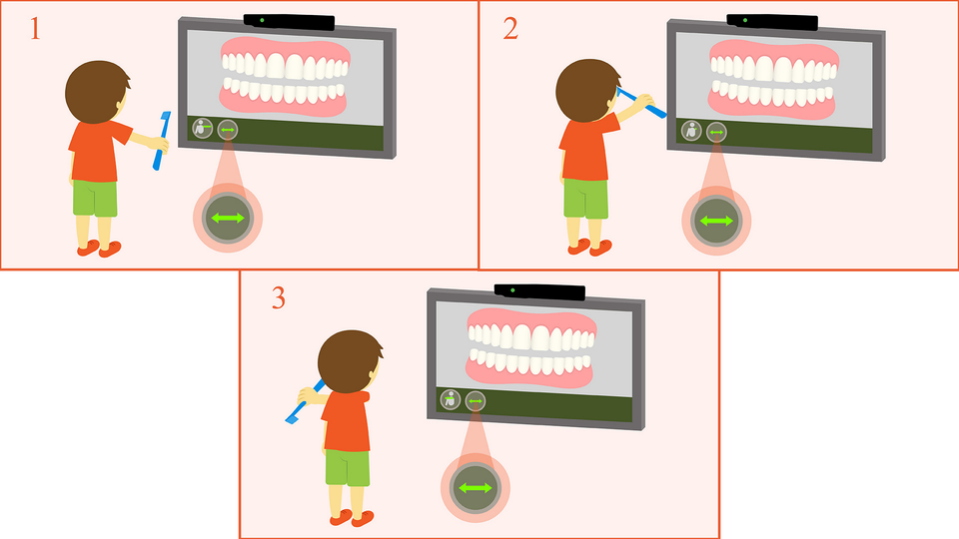
User interaction in the serious game with Kinect using the Stillman brushing technique.

For movement capture, the Kinect sensor uses a programming code that processes its reading and sends the data in a specific format to the system. The player’s movement while performing the toothbrushing technique in the expected way will be detected. Then, after four successful repetitions of valid toothbrushing positions and movements, the user will be directed to another screen to execute the same movements, this time with the character’s mouth in a frontal position. The toothbrushing movements must be repeated both for the maxillary and mandibular teeth. Figures 5 and 6 show the interaction between user and serious game using Kinect. Through the integration of Unity 3D and Kinect, it will be possible to effectively capture the movements of Fones and Stillman toothbrushing techniques and send them to the game, in real time, on the screen.

**Figure 7:**
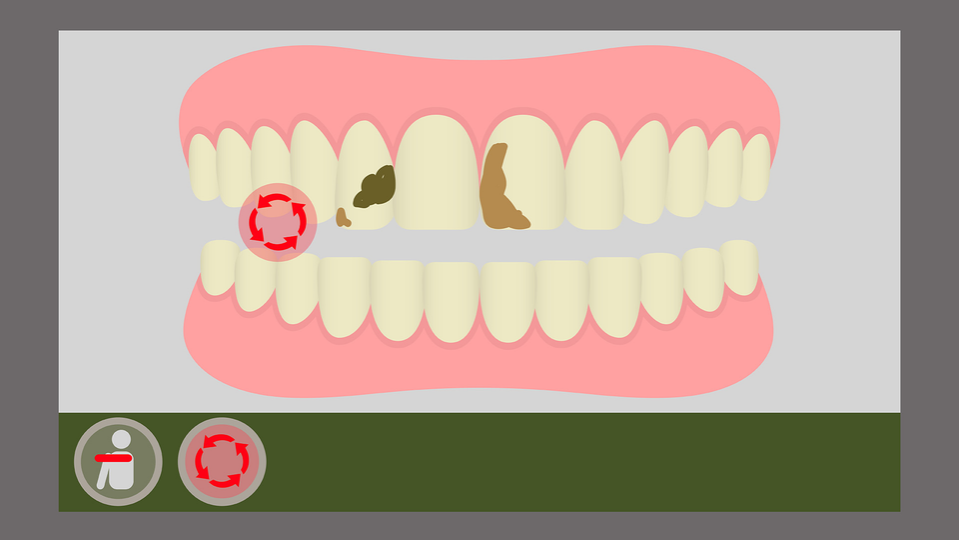
Indication of the Fones technique movement.

**Figure 8:**
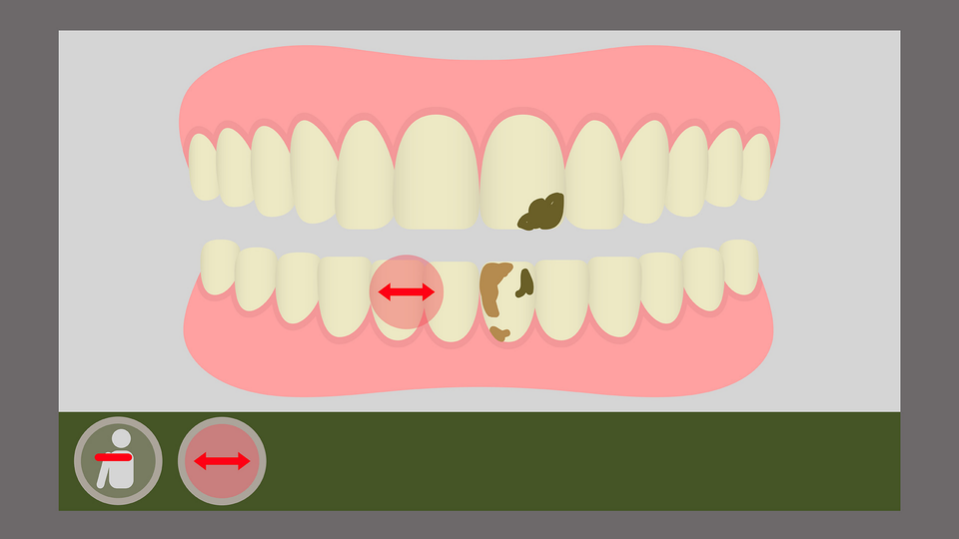
Indication of the Stillman technique movement.

The game has also an error identification implementation. Whenever the player executes the chosen brushing technique incorrectly (ie, if the player does not reproduce the expected movements in the correct speed, time, and angle), the system provides a visual response with an on-screen indication on the avatar’s mouth region. An arrow indicates the format, speed, and angulation of the movement the player should have been executing (Figures 7 and 8).

**Figure 9:**
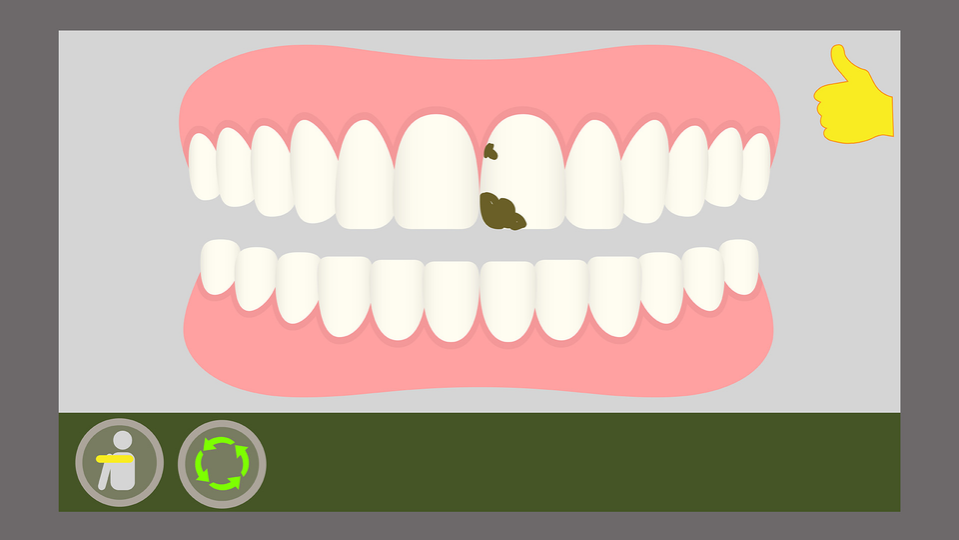
On-screen representation of the Fones movement with residue fixation.

**Figure 10:**
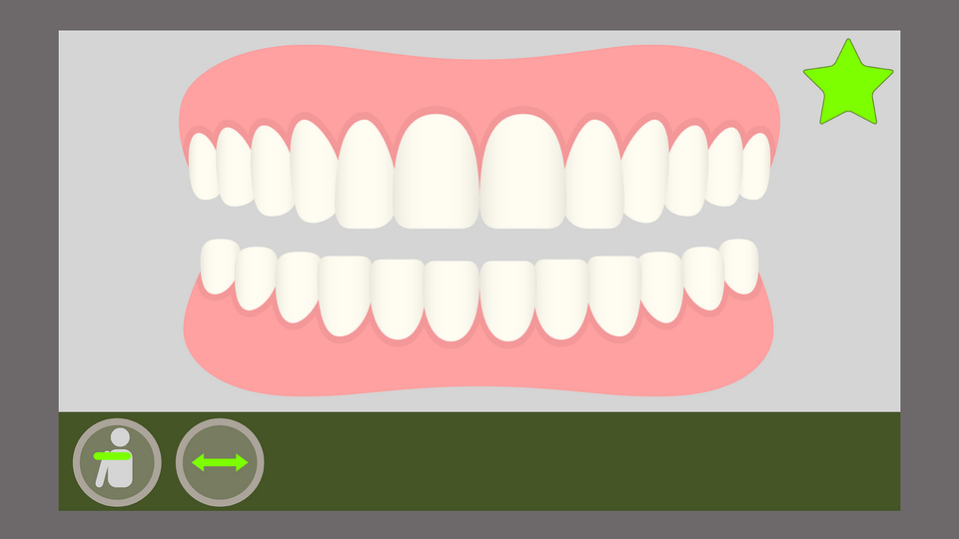
On-screen representation of the Stillman movement with total residue removal.

Furthermore, the game provides a metric for an effective toothbrushing. For this purpose, several challenges are presented to the player as food residues represented graphically and in a ludic way on the character’s tooth surface. On the scene, the player must remove the residues by reproducing the toothbrushing movements. Therefore, not only must the child execute these chosen toothbrushing technique movements correctly, they must also reproduce these movements on the sites (movement angle, height, and amplitude) where the residues are located to remove them completely. This is measured by means of a scoring system: if the player only executes the movements but does not remove the residues, they will receive a lower score at the end of the game; if the player manages to remove all residues, the score will be higher, according to the amount (partial or total) of elements removed during the toothbrushing (Figures 9 and 10).

## Results

This study protocol defines the use of AR on oral hygiene practice by means of an interactive interface that makes use of the Kinect movement sensor. The training of toothbrushing techniques will become more feasible, effective, and accessible to children. After the implementation of the virtual interactive and immersive panels, enrollment will begin and evaluations will be made by means of questionnaires distributed to participants who interacted with the game. Thus, an analysis of product efficacy will be conducted. The expected outcome is to provide a digital instrument to improve oral hygiene practice in children and also motivate the prevention of oral diseases by sharing scientific research in the school environment and community.

## Discussion

This study presents the development protocol of an instructional immersive interactive environment using projection and computational resources. From a methodological and technological point of view, the innovation of this tool will allow the effective interaction of the target audience. The serious game will be able to be edited for updates and changes, making this tool an instrument of scientific teaching and research on different levels of learning. New technologies create possible new ways of action, interaction, and learning, which is relevant in the field of education. It is worth highlighting that digital design applied to health education is a subject still little explored but with a significant and relevant potential for social contribution and improvement of oral health. There is a lack of protocol in using an immersive interactive ludic-educational interface to motivate oral hygiene practice in children by means of AR.

The process of dissemination and consolidation of the use of technology applied to education and health increases the use of digital technologies. This digital access led to the birth of generations with visibly distinct characteristics regarding information, the so-called “digital natives” and “digital immigrants” [15]. These terms are used to describe, respectively, individuals who were born immersed in digital technologies and individuals who were raised with no contact with such technologies. This generation gap was identified [16] and discussed throughout the first decade of this century as a challenge to be faced by the fields of resources and professional development in the educational area. The new generation of digital natives is looking for new content and activities that might encourage them to study and learn.

The virtual realism may be associated with static or dynamic objects [17]. In the training of this applied study, users will be able to visualize and interact in a virtual environment, feeling it as real; that is, the more immersion the simulator is able to provide, the more realistic it will be considered [17].

After the implementation of the virtual interactive and immersive panels, the concept proof will be applied (ie, technical and computational performance tests that evaluate the functionality requisites). The concept proof aims to enable the best performance during the simulation, within the specific characteristics of realism of the 3D realistic virtual model. After that, the application and use of the product will be validated and the learning object applied in other projects.

## Abbreviations

AR: augmented reality
